# The molecular evolution of the vertebrate behavioural repertoire

**DOI:** 10.1098/rstb.2015.0051

**Published:** 2016-01-05

**Authors:** Seth G. N. Grant

**Affiliations:** Centre for Clinical Brain Science, Edinburgh University, Chancellors Building, 49 Little France Crescent, Edinburgh EH16 4SB, UK

**Keywords:** synapse, MAGUK, genome duplication, cognition, GluN2, Dlg

## Abstract

How the sophisticated vertebrate behavioural repertoire evolved remains a major question in biology. The behavioural repertoire encompasses the set of individual behavioural components that an organism uses when adapting and responding to changes in its external world. Although unicellular organisms, invertebrates and vertebrates share simple reflex responses, the fundamental mechanisms that resulted in the complexity and sophistication that is characteristic of vertebrate behaviours have only recently been examined. A series of behavioural genetic experiments in mice and humans support a theory that posited the importance of synapse proteome expansion in generating complexity in the behavioural repertoire. Genome duplication events, approximately 550 Ma, produced expansion in the synapse proteome that resulted in increased complexity in synapse signalling mechanisms that regulate components of the behavioural repertoire. The experiments demonstrate the importance to behaviour of the gene duplication events, the diversification of paralogues and sequence constraint. They also confirm the significance of comparative proteomic and genomic studies that identified the molecular origins of synapses in unicellular eukaryotes and the vertebrate expansion in proteome complexity. These molecular mechanisms have general importance for understanding the repertoire of behaviours in different species and for human behavioural disorders arising from synapse gene mutations.

## 1. A brief historical introduction to the behavioural repertoire

The notion that humans and other animals use a behavioural repertoire of individual behavioural responses was articulated in the nineteenth century by Herbert Spencer, Charles Darwin, George Romanes and William James. In his *Principles of Psychology*, James described components of the behavioural repertoire of humans including sensations, instincts, memory, perception, imagination, reasoning and emotion among others [1]. Individual components, or combinations of components, were available for the exigencies arising in the course of quotidian life.

Romanes and James wrote extensively on the relationships between reflexes, instincts and higher forms of cognition. Indeed Romanes, a protégé of Darwin and considered as the father of evolutionary psychology, suggested that there was a hierarchical continuum between these three broad classifications of behaviour. These pioneers noted that invertebrates and even unicellular organisms displayed reflexes, instincts and a capacity to learn. Indeed, Charles Sherrington, who is principally known for his electrophysiological studies of the reflex, recognized that the protozoa *Vorticella* exhibited all three major components of the reflex (reception, conduction, end-effect) [2]. He noted that metazoans had specialized these three components of the reflex into individual cell types and specific structures. These scientists recognized that simple or unitary behaviours, such as the reflex, may be building blocks for much more complex behaviours. James wrote ‘The actions we call instinctive all conform to the general reflex type’ and the ‘nervous system is to a great extent a preorganized bundle of such reactions'. These foundations have underpinned much of our current thinking on the cellular basis of behaviour [3].

These writings suggest the model that the ancestral basis for the diverse behavioural repertoire of humans, and other vertebrates, may be found in unicellular organisms, and through the process of specialization and adaptation, the ancestral mechanisms derived novel functions that underpin the various components of the behavioural repertoires of metazoans. As to the identity of the molecular and cellular mechanisms, the nineteenth century scientists could only draw upon a limited amount of data. Nevertheless, they recognized that anatomical specialization was important (e.g. afferent and efferent nerves of the reflex arc), as well as neuronal activity and hormonal effects.

In the aftermath of Darwin's theory of evolution, there was intense interest in the relationships between the complexity of human behaviour and that of other animals. There was a broad consensus that invertebrates and vertebrates shared reflexes and instincts, but vertebrates were endowed with more sophisticated and diverse components contributing to their ‘higher’ cognitive repertoires. How could vertebrates have evolved a more complex set of behaviours? The dominant hypothesis is that the greater size of the vertebrate nervous system (increased numbers of cells and synapses) and brain regional specialization is the key determinant. However, this hypothesis has remained untested and therefore unproven. Thus, a major question in biology that remains is: how did the mammalian vertebrate behavioural repertoire evolve and what were the underlying mechanisms?

## 2. The centrality of synapse proteins in the behavioural repertoire

Synapses, which are the hallmark of the brain, appear to play a fundamental role in the repertoire since both pharmacological and genetic interference with synapse proteins influences the entire behavioural repertoire: reflexes, instinct, emotions, motor action and cognitive functions. Importantly, these molecular manipulations do not merely show all-or-none effects, but are subtle, with specific changes in different aspects of various behaviours. Thus, there must be specific roles for different synaptic proteins in regulation of components of the behavioural repertoire.

At the electrophysiological level, vertebrate synapses are also remarkably sophisticated, with different activity patterns producing subtle changes in synapse physiology. Indeed, the postsynaptic terminal is the quintessential signal integrator: it receives a highly diverse set of signals in the form of sequences or patterns of pulses of neurotransmitter and it ‘reads' these patterns (also known as the neural code) and then modifies the protein biochemistry and function of the synapse. This activity-dependent modulation is known as synaptic plasticity. Just as behavioural studies have shown subtle roles for different synapse proteins in the behavioural repertoire, different proteins have subtle roles in different aspects of synaptic plasticity. For example, mutations in members of the Dlg family of proteins in mice result in altered forms of long-term potentiation in response to different frequencies of action potentials [4].

## 3. Molecular origins and evolution of synapses

**Figure 1.**
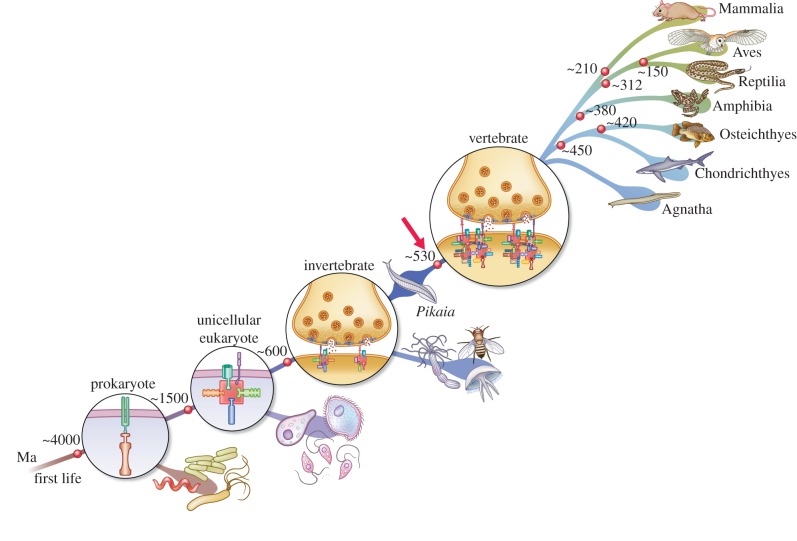
Molecular evolution of the synapse. The postsynaptic proteins comprising the receptor and signalling machinery of vertebrate synapses arose in prokaryotes and eukaryotes and were coopted into the earliest metazoan synapses. The red arrow indicates the two genome duplications that expanded the numbers of proteins to produce the highly complex vertebrate synapse proteome around approximately 550 Ma. The subsequent radiation of vertebrate species is illustrated.

Synapse proteomics has systematically defined the components of synapses and provided the basis for the comparative genomic studies that identified the molecular origins of synapses [5–7]. As synapse proteins play a role in all aspects of the behavioural repertoire of metazoans, it might seem unlikely that synapse signalling mechanisms could be relevant to the behaviour of unicellular organisms. However, it is now known from comparative genomics of synaptic proteins that all of the major cell-biological processes of the pre- and postsynaptic terminal evolved in unicellular eukaryotes and that many of these proteins and pathways arose in prokaryotes [5–7]. These include the most important mechanisms of neurotransmitter release and response—the vesicular release machinery and postsynaptic proteins that mediate synaptic transmission and plasticity. Hence the synapse, which is the centrepiece of the metazoan brain, is built from molecular constituents that first evolved in unicellular organisms. It follows that these ‘protosynaptic mechanisms' were coopted into the first nervous systems of metazoans. The molecular evolution of the synapse is summarized in figure 1 and reviewed in detail elsewhere [5,7].

In addition to revealing the molecular origins of synapses, comparative genomics and proteomics have also discovered major differences between invertebrate and vertebrate synapses. While humans, mice and many other vertebrates share very similar numbers and types of synapse proteins, *Drosophila* and other invertebrates showed fewer proteins, albeit in the same classes as those in mammals [6,8]. This has been referred to as the ‘vertebrate expansion’ in synapse proteome complexity. In 2008, comparative genomic studies provided evidence that two whole genome duplications (2WGD) early in the vertebrate lineage (approx. 550 Ma) created, from ancestral genes, many new members of the extant gene families of vertebrates [9,10]. The 2WGD events appear to be the driver for the ‘vertebrate expansion’ in synapse proteome complexity.

## 4. A theory of vertebrate behaviour based on synapse gene evolution

My colleagues and I have hypothesized that the greater complexity in the vertebrate behavioural repertoire and synaptic plasticity arose from the expansion and diversification in synapse proteins [11,12]. Or to put it more simply, vertebrate behavioural complexity is a product of synapse proteome complexity. Central to this hypothesis is Ohno's exposition of the importance of gene duplication in creating new functions [13]. He proposed a general model where the creation of new gene copies permits a relaxation of constraint on the sequence of the duplicated gene(s) and hence new functions are derived. As a result, paralogues may share (conserved) ancestral functions or have novel (derived) functions. To test the hypothesis, it was necessary to study paralogues of synapse proteins in behaviour and physiology.

Here I will overview and synthesize three published experiments that address different aspects of the mechanisms of gene duplication in synaptic proteins. These three experiments will be framed within the context of three temporally distinct events in the molecular evolution of synapses during the last approximately 550 Myr: the 2WGD events that generated paralogues, the diversification in the paralogues' sequences that occurred during the next 50–150 Myr, and finally, the period of constraint where paralogue sequence diversification was reduced in the last approximately 90 Myr (in mammalian evolution). These experiments exploit two gene families that encode proteins of fundamental importance to synapse function: the NMDA receptor and the Dlg/membrane-associated guanylate kinase (MAGUK) scaffold proteins. Members of the gene families were genetically engineered in mice to alter their functions and the mice were tested in behavioural and electrophysiological assays. Importantly, a wide range of behavioural components were tested, using a battery of apparatus, so as to quantify the effects on a repertoire or set of behaviours. The analysis of these data enables one to examine the role of synapse proteome expansion, gene duplication and many aspects of molecular evolution in behaviour. These are the first studies of the genetic dissection of multiple components of a broad behavioural repertoire.

## 5. Genome engineering approaches to testing synapse protein evolution

**Figure 2.**
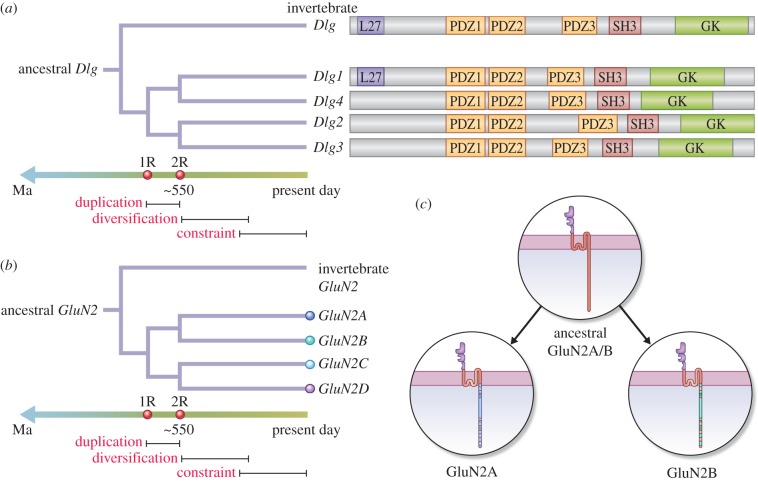
Two genome duplications generated paralogues of *Dlg* (*a*) and *GluN2* (*b*) genes. Both (*a,b*) show timelines indicating the relative proximity of the duplication, diversification and constraint phases. Trees show single ancestral genes resulted in four paralogues after two duplication events. The period of gene duplication, diversification and constraint is illustrated. The first round of duplication (1R) and second round (2R) are illustrated on the timeline. Linear diagrams of Dlg1–4 proteins with protein domains (L27, PDZ, SH3, GK) are also shown in (*a*). (*c*) The ancestral GluN2 subunit diversified predominantly in the cytoplasmic terminal domain (CTD) of GluN2A and GluN2B. Adapted from Ryan *et al.* [14].

As shown in figure 2, the importance of duplication, diversification and constraint was addressed by studies of the paralogues in two gene families: the Dlg/MAGUK proteins (*Dlg1*/SAP97, *Dlg2*/PSD93, *Dlg3*/SAP102, *Dlg4*/PSD95) (figure 2*a*) and the GluN2 subunits of the NMDA receptor (*GluN2A* and *GluN2B*) (figure 2*b*). Paralogues in these families were genetically manipulated in mice, and these mice were used in behavioural tests that probed components of their behavioural repertoire.

These gene families were chosen because (i) previous genetic and pharmacological studies show they are of paramount importance in synaptic plasticity and cognition and among the most widely studied of synaptic proteins, (ii) they represent two distinct classes of functionally important proteins (*GluN2* genes encode neurotransmitter receptor subunits and the Dlg/MAGUK proteins are cytosolic scaffolding proteins that bind to GluN2), (iii) these two families of proteins are known to bind and assemble into protein complexes (called MASCs, MAGUK-associated signalling complexes) [15,16], and (iv) their gene structure permits specific types of genetic engineering, which is suitable for addressing gene duplication, diversification and constraint.

The function of four *Dlg* paralogues (*Dlg1, Dlg2, Dlg3, Dlg4*) was compared by using lines of mice carrying null alleles in each of the genes (figure 2*a*) [11]. This enabled a straightforward comparison of the behavioural phenotype of each gene. As this strategy does not enable one to distinguish the conserved and derived functions that could exist between a pair of paralogues, a second complementary and more sophisticated genome engineering approach was also adopted by focusing on the NMDA receptor subunits [12], GluN2A and GluN2B, which show highly conserved sequences in their extracellular and membrane spanning domain but low similarity in their cytoplasmic C-terminal domains (CTD) (figure 2*b,c*) [14]. The exon encoding the CTD from GluN2A was removed and replaced with the corresponding exon encoding the GluN2B CTD, and vice versa (these mice were referred to as ‘swap mice’) [12]. Thus, any functions that were conserved between GluN2A and GluN2B will be unaffected by this manipulation, whereas those derived functions in each paralogue will result in altered phenotypes. These two complementary genetic engineering strategies in two classes of synaptic proteins underpin a comprehensive test of the hypothesis that duplication and diversification in vertebrate synapse proteins regulated components of the cognitive repertoire.

## 6. The role of gene family duplications in the vertebrate behaviour repertoire

**Figure 3.**
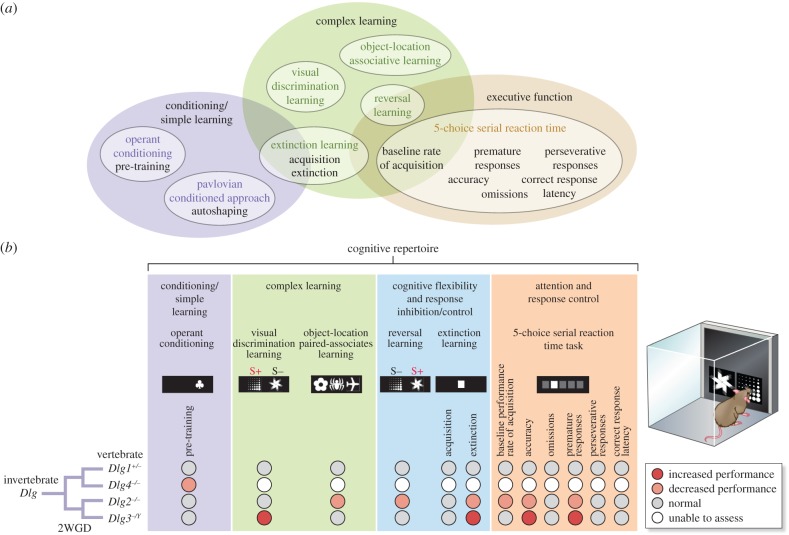
*Dlg* paralogue function in the mammalian cognitive repertoire. (*a*) The components of the mammalian cognitive repertoire divided into three broad categories (purple, green and brown ellipses) within which the specific measures and tests are labelled. See Nithianantharajah *et al.* [11] for details. (*b*) Behavioural phenotypes of four *Dlg* mutant mouse lines in 12 measures with four cognitive domains. Left shows tree indicating duplications from an ancestral *Dlg* gene. Simple learning required *Dlg4*, but not *Dlg2* or *Dlg3*. *Dlg2* and *Dlg3* had opposing phenotypes in extinction learning, accuracy and premature responses. Adapted from Nithianantharajah *et al.* [11].

In the study of the *Dlg* paralogues, Nithianantharajah *et al*. [11] examined components of the behavioural repertoire (figure 3*a*) using computerized touchscreen methods that are now widely used to measure components of cognition in humans and rodents [17]. Mice are rewarded with food after touching their nose to images presented on the touchscreen. A range of test paradigms have been developed that together assess attention, perception, simple and complex forms of learning and executive functions (figure 3*b*) [18–20]. These tests have been organized into a battery of 12 primary measures (shown in figure 3*b*). Mice from each of the four lines of mutants carrying null alleles of *Dlg1–4* genes were tested in this battery. As shown in the summary figure 3*b*, each *Dlg* paralogue showed a specific profile of behavioural changes in the 12 measures, indicating that each gene had evolved specific function in shaping the behavioural repertoire.

Beyond identifying differences between the phenotypes of paralogues, there were further insights into the role of the 2WGD events. On the basis of sequence comparisons, the first genome duplication generated genes that were the ancestors of *Dlg1/4* and *Dlg2/3*, respectively (illustrated on the left of figure 3*b*). *Dlg1* has the greatest homology to the invertebrate *Dlg* gene. Interestingly, *Dlg1* and *Dlg4* showed the most severe phenotypes: *Dlg1* knockouts were non-viable (behaviour was studied in heterozygous mice and showed no phenotypes) and *Dlg4* mice were impaired on the simplest forms of learning and were incapable of all complex forms of learning. By contrast, *Dlg2* and *Dlg3* knockouts had no impairments in simple learning but were required exclusively for complex forms of learning and attention. This suggests that the first genome duplication permitted separation of functions of these two pairs of paralogues. Moreover, comparison between *Dlg2* and *Dlg3* shows a dichotomy where each has an opposing effect on extinction learning and components of attention (*Dlg2* showed decreased performance and *Dlg3* increased performance). This indicates that each of these two paralogues has evolved specialized functions after the second genome duplication event, resulting in greater behavioural response control, effectively tuning and counterbalancing these key components of the behavioural repertoire. A parsimonious model is that *Dlg4* retained an ancestral (invertebrate) function in simple forms of learning, whereas the diversification of *Dlg2* and *Dlg3* provided novel regulation of complex cognitive processes arising in vertebrates. Together these results show that paralogue diversification has provided gene-specific regulation of components of the vertebrate cognitive repertoire, hence contributing to vertebrate cognitive complexity.

## 7. Diversification of paralogue protein sequence in evolution of behaviour

As noted above, the strategy of comparing null alleles that was used in the study of *Dlg* paralogues provides limited information on those behaviours that rely on conserved or ancestral (prior to the duplication) gene sequences and those behaviours that rely on derived (after the duplication as a result of sequence diversification between paralogues) gene functions. Ryan *et al*. [12] devised a strategy aimed at distinguishing those behaviours controlled by conserved and derived functions in a pair of paralogues in the *GluN2* family. This strategy takes advantage of the GluN2A and GluN2B subunit's CTDs that are widely divergent (29% sequence conservation at amino acid level) [14] (figure 2*c*). Hence it is possible that the CTD of each paralogue retained ‘ancient’ roles in regulating certain behaviours and, as a result of diversification in protein sequence, each CTD may have also gained or lost regulation of other specific behaviours.

**Figure 4.**
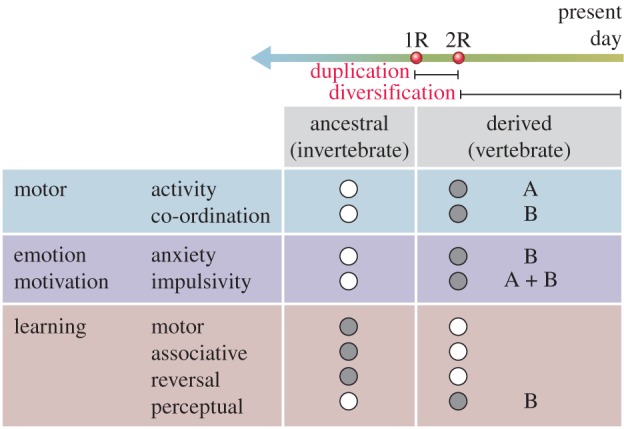
Diversification in GluN2A and GluN2B CTD regulation of the behavioural repertoire. Eight behaviours grouped into three broad domains—motor function, emotion and motivation, learning and memory—were classified into those that were regulated (shaded circles) by ancestral (invertebrate) sequences conserved in GluN2A and GluN2B CTDs, and those that were regulated by derived functions in the CTDs. The letters (A or B) in the derived functions refer to the specific GluN2A and GluN2B subunits' roles. The timeline illustrates that the ancestral or conserved sequences date prior to the duplications (1R and 2R) and the derived function arose after the duplications.

Using a behavioural test battery comprising touchscreens, open field, elevated plus maze, novel object recognition, fear conditioning and rotating rod apparatus, Ryan *et al.* identified a repertoire consisting of eight behaviours, all of which required the function of the NMDA receptor [12]. These behaviours spanned cognitive functions and motor functions as well as emotions/anxiety. To tease out which of those behaviours were regulated by conserved and derived functions, they compared the phenotypes of the swap mice and two other lines of mice carrying loss-of-function mutations in *GluN2A* and *GluN2B*. As summarized in figure 4, the eight behaviours, grouped into three broad domains—motor function, emotion and motivation, learning and memory—were genetically dissected. It was deduced that motor, associative and reversal learning were all regulated by conserved protein sequences in (both) GluN2A and GluN2B CTDs. By contrast, the other five behaviours had evolved specific regulation after the diversification of the CTD sequences. Some behaviours were specifically regulated by protein sequence in GluN2B CTD and others by GluN2A: GluN2A CTD regulated locomotor activity and impulsivity and GluN2B CTD regulated perceptual learning, anxiety, impulsivity and motor coordination, which must have arisen as a result of protein diversification after the duplication event approximately 550 Ma.

The authors further interpreted the data to suggest how vertebrate behaviours may have evolved. They suggested that the adaptive value of gene evolution in behaviour lies in the changes to the overall repertoire and not in any single phenotype. On the basis that the only behaviour regulated by diversification in both GluN2A and GluN2B was impulsivity, and that anxiety and motor activity required a unique amino acid sequence from either the GluN2A or GluN2B CTD, they also suggested that the protein sequences controlling emotional and motivational behaviour were less constrained by natural selection than those sequences that regulate learning behaviours, as three of the four learning behaviours did not require an amino acid sequence unique to either GluN2A or GluN2B. Hence they postulated that greater regulation of emotional and motivational behaviour conferred an adaptive advantage on early vertebrates. They also noted that learning, emotional and motor behaviours are fundamental animal behaviours that can be observed in simple forms, even in invertebrate species, and that these behaviours acquired distinct forms of regulation during vertebrate evolution. This is consistent with the conclusion that the overall complexity of the behavioural repertoire increased as a result of these genomic evolution mechanisms, and supports the conclusions obtained (using an orthogonal approach) in the study on *Dlg* paralogues.

## 8. Paralogue constraint in mammalian cognition

**Figure 5.**
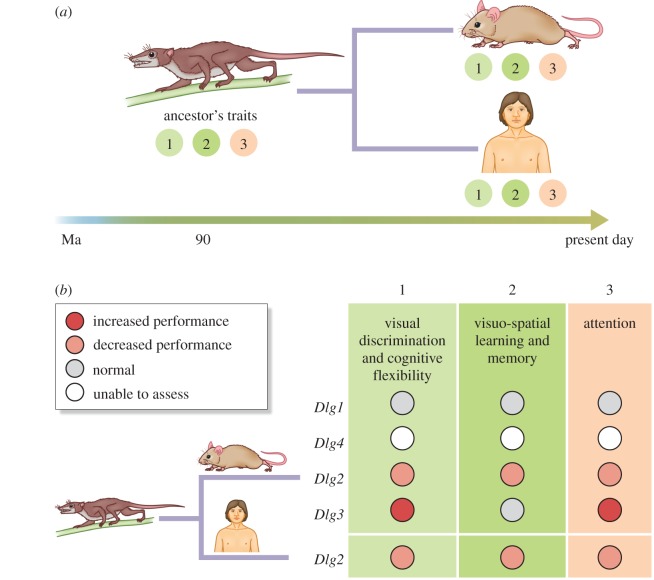
Constraint in *Dlg2* function in humans and mice. (*a*) Humans and mice diverged from a common ancestor approximately 90 Ma and three hypothetical conserved behavioural traits/components are illustrated. (*b*) Results of touchscreen tests of three components of cognition (1, visual discrimination and cognitive flexibility; 2, viso-spatial learning and memory; 3, attention) are illustrated for four lines of *Dlg* mutant mice and human carryings *Dlg2* mutations. Note the conserved cognitive phenotypes of human and mouse *Dlg2* indicating that this gene's regulation of cognition has been conserved from the common ancestor of humans and mice.

I will now discuss experiments that address the third phase—the period after diversification of paralogues, when sequence constraint limited their further diversification. It has been estimated that most sequence diversification occurred within 50–150 Myr of the duplication events in the vertebrate lineage [21]. Consistent with this, there is a high degree of homology between human and mouse synaptic proteins (e.g. more than 95% similarity in protein coding of *Dlg* orthologues), which diverged from a common ancestor approximately 90 Ma [11,22,23]. To assess whether the function of a *Dlg* gene in regulating multiple components of the behavioural repertoire was maintained after the divergence of humans and mice from their common ancestor (figure 5*a*), Nithianantharajah *et al*. [11] compared the results of touchscreen tests of cognitive behaviours in humans and mice carrying *Dlg2* mutations. They found that both humans and mice carrying *Dlg2* mutations showed impairments in the same components of the cognitive repertoire (figure 5). The authors also compared gene expression patterns in the mouse and human brain and identified conserved brain regional expression of *Dlg* paralogues. Together these results indicate that the conservation and constraint at the genomic level has maintained these gene-to-cognition relationships between the two species, and that the genetic architecture of mouse and human cognitive repertoires share common synaptic mechanisms.

## 9. The repertoire of behaviour

These behavioural genetic experiments have tackled a fundamental question in biology: how did the vertebrate behavioural repertoire arise and evolve. They show, for the first time, an experimental proof of a mechanism explaining the mechanisms underlying the complexity and diversity of vertebrate cognition and other behaviours forming the repertoire [24]. The genome evolution that produced the paralogues and expanded synapse proteome complexity has contributed complexity to the behavioural repertoire of vertebrates. This can be encapsulated as the synapse proteome expansion theory of vertebrate behavioural complexity.

The synapse proteome expansion theory of vertebrate behavioural complexity has many implications for behaviour. In addition to *Dlg* and *GluN2* paralogues, it is expected that paralogues within many other synaptic proteins will follow similar principles. The expansion in the synaptic proteome could contribute to the evolution of many new subtle aspects of the behavioural repertoire, as suggested by the comparisons of the *Dlg* paralogue phenotypes. The ‘simple’ ancestral invertebrate behaviours could have acquired more sophisticated molecular regulatory mechanisms and therefore the vertebrate behaviours have finer, more subtle tuning. The expanded and more flexible set of cognitive functions may have implications for explaining the range of environmental niches into which vertebrates have adapted. Subtle lineage-specific genetic variation in synapse proteins could give rise to tuning or modifications in particular components of the behavioural repertoire relevant to an environmental niche. For example, the *Dlg* and *GluN2* genes regulate the amount of time a mouse spends in the elevated open arm of a maze (where they are anxious about being exposed and falling) and if there was sequence variation in these genes between species, this particular behaviour could be tuned accordingly. Such mechanisms might be relevant to the differences between species such as ungulates where some species (mountain goats) dwell on precipices whereas others dwell on plains (antelopes). Hypotheses regarding the role of gene function and environmental niches could be tested by at least two approaches: the *Dlg* and *GluN2* mutant lines of mice could be studied in more ethologically relevant environments, and genetic variants identified in other species could be engineered into the mouse genome for later behavioural testing.

The combinatorial action of duplicated genes is another mechanism that shapes the behavioural repertoire of vertebrates and has a direct link to synapse proteome complexity. An intrinsic feature of genome duplications is the multiplicative complexity that arises from the action of combinations of genes. To illustrate this, consider an ancestral receptor assembled from four subunits, each encoded by a single gene, and after 2WGDs there would be 16 genes that could be organized into a very large number of types of receptor. As the number of subunits or components in multiprotein complexes or pathways increases, the potential for vast multiplicative complexity and diversity arises from genome duplications. It is therefore important to identify the paralogue specializations that reduce this complexity. In this context, a clustering analysis of behavioural components and *Dlg* paralogues showed specific combinations of *Dlg* genes were required for specific behaviours [11,25]. Surprisingly, behaviours that were considered to be similar (two forms of complex learning or three forms of cognitive flexibility) were genetically separable by specific combinations of *Dlg* mutations. Thus, there is not simply a redundant and promiscuous use of paralogues in the specification of components of the behavioural repertoire. Little is known about the derived restrictions and specializations that were responsible for controlling the behaviour repertoire and limiting the complexity explosion. In addition, these findings suggests a mechanism by which ancestral behaviours, regulated by an ancestral gene, can give rise to a set of related, but independently regulated, derived forms of that behaviour.

In addition to the role of genome duplications in synapse proteome expansion, alternative splicing also generates diversity within protein isoforms. Alternative splicing is highly abundant in the central nervous system of vertebrates [26–28]. Interestingly, the duplicated Nova splicing factor genes (Nova1 and Nova2) have derived roles that differentially regulate the neuronal transcriptome with Nova2 demonstrated to regulate the synapse proteome [29]. The alternative splicing will add another layer of multiplicative complexity to the molecular diversity in the vertebrate synapse proteome.

Octopus are cephalopods with large nervous systems and among invertebrates are considered to have complex behaviours [30,31]. They are known to have remarkably sophisticated motor skills, ability to discriminate and several forms of learning and memory. The recent sequencing of the octopus genome identified several *Dlg* paralogues, which likely arose by gene rather than genome duplication [32]. Although it has been demonstrated that the synapse proteome of *Drosophila* is less complex than that of mouse synapses [6], it will also be important in future to study the synaptic proteome complexity of octopus directly. These studies will enable one to determine whether the *Dlg* genes in octopus encode synapse proteins and whether there are other synapse proteins that have expanded. The physiological and behavioural functions of octopus Dlg proteins remains unknown, although it is perhaps likely that the *Dlg* paralogues diversified the organization of the protein complexes in octopus synapses and generated multiple forms of synaptic plasticity, as observed in mice [4,11]. The recent advances in CRISPR/Cas9 genome engineering methods, which have been adapted for use in insects [33,34], worms [35] and *Ciona* [36], hold out the prospect that direct testing of the role of duplicated *Dlg* genes in octopus will be feasible, although the techniques for introducing materials into octopus eggs await development. It may even be possible to perform exchange of paralogue domains at the genomic level, as has been done in mice [12]. These types of genome engineering experiments will need to be coupled with the appropriate quantitative tests of the components of the octopus behavioural repertoire. A complementary approach is to engineer the invertebrate gene sequences into the mouse.

Synapse proteome expansion in vertebrates also provides a perspective on anatomical complexity. The vertebrate expansion of synapse proteins was shown to result in differential distribution of synaptic proteins in different brain regions in mice and humans [6,37]. Importantly, the 2WGD events that produced the expansion in vertebrate synapse proteomes occurred prior to the anatomical diversification in many brain regions and encephalization that characterizes the tetrapod brain. This is significant because until now, most hypotheses regarding the behavioural sophistication of vertebrates have assigned primacy to anatomical explanations, typically based on numbers of nerve cells or connections [38]. However, primacy should be assigned to the genome duplication events and synapse proteome expansion. This allows new hypotheses to be postulated regarding the importance of brain size and anatomical specialization. For example, given that synapse proteome expansion was also accompanied by diversification in gene regulation and hence diversification in anatomical expression patterns of synapse proteins, it is likely that synapse diversity and concomitant neuronal and brain regional diversity is a secondary consequence of 2WGDs. In other words, synapse proteome expansion generated diversification of synapse types and these will be distributed across the nervous system. Indeed, not only are *Dlg* and *GluN2* paralogues differentially distributed, but so are many of the other synaptic paralogues [6,37]. These new models provide fertile areas for future research. Given the extensive literature in comparative anatomy it may be informative to map synaptic proteome diversity of paralogues in species with behavioural and anatomical specializations. It will also be very interesting to genetically modify mice to change the spatial distribution of synaptic paralogues and examine the effects on the behavioural repertoire.

The synapse proteome expansion and its sophisticated behavioural repertoire have come at the price of susceptibility to mental illness, because disease-causing mutations occur in many of these vertebrate paralogues. For example, *Dlg2* mutations result in schizophrenia and *Dlg3* mutations in intellectual disability with autism features [15,23,39–46]. There are hundreds of mutations in postsynaptic proteins that interact with the Dlg and GluN2 proteins in the postsynaptic density [23]. These mutations also reveal the subtlety of the human behavioural repertoire, which is reflected in disease classifications. For example, it is now apparent that two classifications—autism and schizophrenia—arise from over 100 (polygenic) mutations in synapse proteins.

Behavioural mouse genetics studies have largely overlooked the concept of the behavioural repertoire and have typically focused on specific component of behaviour. It is essential for scientists to develop strategies to examine the behavioural repertoire and further understand its genetic architecture. This is no less important than understanding the genetic architecture of the body plan (bauplan) or the immune response. These key areas of biology have been transformed by the understanding of homeobox genes and immunoglobulin gene structure, respectively, and both have genetic mechanisms that have been powerfully shaped and underpinned by gene and genome duplication events.

Finally, we can revisit the insights of the nineteenth century pioneers who drew connections between synapse mechanisms, general features of behaviour including continuums from simple to complex behaviours, and shared mechanisms found in many life forms. A molecular understanding of the building blocks of the behavioural repertoire may lead to unifying theories of behaviour. The postsynaptic mechanisms appear to be such building blocks and with new methods of experimental genetics there is an exciting new prospect for many ethological and laboratory studies of behaviour in a wide range of species.
